# Challenges prescribing and dispensing oral antibiotics with poor palatability for paediatric patients: A qualitative interview study with GPs and pharmacists

**DOI:** 10.1016/j.rcsop.2024.100546

**Published:** 2024-11-23

**Authors:** Ayat Elgammal, Joseph Ryan, Colin Bradley, Abina Crean, Margaret Bermingham

**Affiliations:** a Pharmaceutical Care Research Group, School of Pharmacy, University College Cork, Cork, Ireland; b SSPC Pharmaceutical Research Centre, School of Pharmacy, University College Cork, Cork, Ireland; c Department of General Practice, School of Medicine, University College Cork, Cork, Ireland

**Keywords:** Antibiotics, Palatability, Caregivers, Paediatrics, Patient adherence, Antimicrobial resistance, Primary care

## Abstract

**Background:**

Poor palatability of antibiotics is a key cause for non-adherence to antibiotic treatment among children. Failure to complete antibiotic treatment because of poor palatability can cause disease recurrence and may contribute to increasing rates of antimicrobial resistance. The aim of this study was to investigate the experience and challenges faced by general practitioners (GPs) and community pharmacists regarding prescribing and dispensing oral liquid antibiotics for children and the impact of poorly palatable antibiotic formulations on patients and the health-system.

**Methods:**

One-to-one semi-structured interviews with GPs and pharmacists were conducted via an online video-conferencing platform. Data were analysed using thematic analysis.

**Results:**

Twenty participants (7 GPs and 13 pharmacists) were interviewed. Three main themes and eight subthemes were identified. Theme 1: *challenges reported by GPs and pharmacists* included four subthemes; (i) factors affecting prescribing and dispensing antibiotics, (ii) reasons to select poorly palatable antibiotics, (iii) palatability discussion with parents, and (iv) formulation factors affecting oral liquid antibiotic acceptability. Theme 2: *the impact of prescribing or dispensing poorly palatable oral liquid antibiotics* encompassed two sub themes; (i) patient impact and (ii) health-system impact. Theme 3: *overcoming palatability challenges* involved two subthemes; (i) raising awareness of flavour and palatability issues among healthcare professionals and (ii) counselling parents while prescribing and before dispensing.

**Conclusions:**

There is a need to increase palatability awareness among healthcare professionals and parents. The development of more palatable oral liquid formulations can play a role in improving prescribing and medicines taking practices.

## 1 Introduction

Children's age, weight and potential allergies can influence choice of medicines for this patient group. However, children can also experience sensory-related problems such as issues with taste, smell, and texture when administered oral prescription medicines.^1^^,^^2^ Antibiotics are one of the most frequently prescribed medicines for paediatric patients.^3^, ^4^, 5. However, poor palatability, based on smell, taste, aftertaste, and texture of antibiotics is one of the key causes for non-adherence to antibiotic treatment among paediatric patients.^6^, ^7^, 8. Failure to complete antibiotic treatment because of poor palatability can cause disease recurrence and can result in switching to a more palatable antibiotic which is typically a more broad-spectrum antibiotic.^9^^,^^10^ This can lead to the development of antimicrobial resistance and may play a role in expanding rates of antimicrobial resistance in the general population.^11^

Young children may experience difficulties in swallowing oral solid dosage forms such as tablets and capsules.^12^ Therefore, a liquid antibiotic formulation is the most common dosage form among children.^13^ A study by Li et al. revealed that two-thirds of the global sales of paediatric antibiotic formulations were in the form of a liquid.^14^ Nevertheless, administration of liquid formulations with poor palatability is a challenge for children where the dissolved drug is in direct contact with the taste buds and olfactory receptors that are more sensitive in children compared with adults.^15^ A study conducted by Venables et al. revealed that several medicines with unacceptable palatability were refused by children or manipulated by parents.^16^ Children's refusal of medicines resulted in spitting out the dose or closing their mouth prohibiting administration. Manipulation through mixing drugs with different foodstuffs or diluting the recommended dose with a flavoured liquid to aid medicines administration, has been suggested as a solution. However, this action can alter the therapeutic response and drug bioavailability.^16^

The clinical guidelines and availability of supply were ranked as the most important factors affecting the selection of antibiotics by the majority of GP and pharmacist respondents respectively in a recent survey conducted by the authors. The survey revealed that a majority of GPs had at times deviated from clinical guidelines, and that the majority pharmacists had changed antibiotic dispensing choice because of palatability issues to ensure treatment adherence. Almost half of respondents of each profession indicated that they rarely or never discussed the palatability with a child's parents or caregivers.^10^ The study presented builds on the results of this prior survey providing a deeper and richer understanding of the survey outcomes. The experience, attitudes, and challenges faced by GPs and pharmacists in primary care regarding prescribing and dispensing poorly palatable oral liquid antibiotics for paediatric patients are investigated and the impact of this issue on patients and health systems.

## 2 Methods

### 2.1 Ethical approval

Ethical approval was granted by the Social Research Ethics Committee of University College Cork, Log no 2021-138A2.

### 2.2 Study design

A topic guide for the semi-structured interviews was developed based on the prior survey responses and associated research literature.^10^^,^^17^ It was further developed through group discussions by the authors. Separate topic guides were used for each profession though the topic guides were broadly aligned. The key difference was that the topic guide for GPs referred to prescribing and the topic guide for pharmacists referred to dispensing oral liquid antibiotics. The topic guide was piloted independently with one GP and one pharmacist in one-to-one interviews. The pilot interviews were conducted to check for length, suitability of the language and potential causes of bias such as leading questions. The pilot interviews were transcribed to check that they have produced enough data related to the research question. Qualitative feedback from the pilot interview were used to refine and finalise the topic guide questions. The pilot interviews were not included as part of the main analysis. The topic guide was agreed by all authors. The topic guide is available as supplementary material.

For contextual background, the study presented was conducted in Ireland where antibiotics are prescribed by GPs and dispensed by pharmacists in Ireland. It is also of note that an Irish pharmacist can switch the medicine brand dispensed (unless the prescriber specifies otherwise on the prescription) and change the strength of a formulation dispensed. Pharmacists cannot change the type of antibiotic dispensed without contacting the prescriber. For oral liquid antibiotic products which require reconstitution, they are reconstituted in the pharmacy prior to dispensing.

### 2.3 Sample characteristics

The inclusion criteria were GPs and trainee GPs registered with the Irish Medical Council; and pharmacists registered with the Pharmaceutical Society of Ireland, who selected community pharmacy as their area of professional practice. Selection of participants was based on obtaining a diverse representation of practitioners such as years of experience, and roles in primary care practice.

### 2.4 Data collection methods

The final survey question in the prior survey of 59 GPs and 185 pharmacists, on the impact of drug palatability on prescribing and dispensing of antibiotic formulations for paediatric patients,^10^ asked participants if they were interested in taking part in a follow-up semi-structured interview. Survey participants who were interested were asked to provide their contact details and were invited to take part in this interview study. The survey and the interview studies were conducted separately to permit in-depth quantitative and qualitative data analysis of the data set obtained from each study. The research team also invoked their professional networks to identify potential participants. These additional participants were invited by email or WhatsApp to take part in the study. A WhatsApp message was sent to professional WhatsApp groups including groups of GP trainees and GP trainers and pharmacist special interest groups of which the research team have membership. Additionally, snowball sampling was invoked, and interview participants were invited to suggest colleagues who might be interested in participating in the study. An information sheet and consent form were sent by email to individuals who indicated an interest in taking part in the study. Participants provided signed, informed consent prior to their interview. Verbal consent was also obtained before starting interviews. One-to-one video-conferencing interviews were conducted using a closed channel of the online meeting platform Microsoft Teams. Each interviewee was provided with a meeting code to guarantee data protection. The semi-structured interviews were conducted by two members of the research team (AE and JR) between December 2022 and December 2023. All GP participants were interviewed by a pharmacist (AE). Pharmacist participants were interviewed by a pharmacist (AE) or a GP (JR).

### 2.5 Data analysis

All interviews were recorded using Microsoft Teams and a transcript was created. The interviews were anonymised and transcribed by AE using Microsoft Word and checked for accuracy by MB and AC. The interviews were then saved in the QSR International NVivo Qualitative Data Analysis Software (V.12) for analysis. Data were analysed using Braun & Clarke's reflexive thematic analysis.^18^ The research team reviewed and familiarised themselves with the interview response data through multiple reading of each manuscript. Elements of interest in the data was inductively coded by AE using conventional content analysis.^19^ To better ensure reliability of coding, samples of transcripts were independently reviewed and coded by MB and AC. Codes were then finalised and grouped into categories related to the study topic. Initial themes representing a number of codes were generated and discussed between all authors. Themes were subsequently refined, and the final themes were agreed by all authors.

### 2.6 Reporting

This study was reported corresponding to the SRQR (Standards for Reporting Qualitative Research) guidelines.^20^

### 2.7 Authors and reflexivity

AE is a pharmacist and PhD researcher. AC, and MB are academic pharmacists in the School of Pharmacy, University College Cork. JR is a GP and CB is an academic GP. All pharmacists and GPs of the research team have experience of dispensing and prescribing oral liquid antibiotics, respectively.

The use of the term parents in this study included caregivers and people who administer antibiotic to children at home.

## 3 Results

### 3.1 Participants

Twenty participants were interviewed, of which seven were GPs, and thirteen were pharmacists. Six participants, three of each profession, were parents themselves. Mean length of interviews was 14 min with a range from 7 to 33 min. The variation in the length of the interviews was related to variability in the style of verbal communication and extent of the detailed responses provided by participants. Sixteen of the participants were female and 4 were male. Both genders were invited to participate in the study. However, a greater number of females agreed to participate. The disparity in gender may somewhat reflect the gender breakdown among registered Irish pharmacists and GPs. The Pharmaceutical Society of Ireland (PSI) and Irish College of General Practitioners (ICGP) annual report reported that 64 % and 56 % of Irish pharmacists and GPs are females respectively. Median years of participants' experience was 11 years with an interquartile range of 2–27 years of practice.

### 3.2 Themes and subthemes

**Fig. 1:**
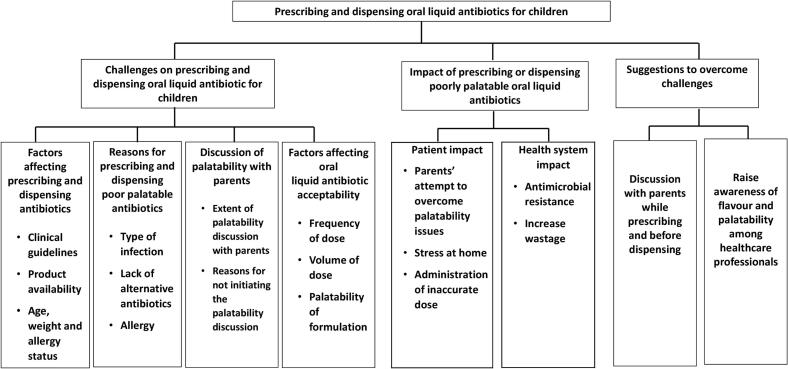
Schematic outlining the identified themes and sub-themes.

Data analysis revealed three main themes and eight sub-themes (Fig. 1). The main themes identified were: (i) the challenges reported by GPs and pharmacists on prescribing and dispensing oral liquid antibiotic for paediatric patients, (ii) the impact of prescribing or dispensing poorly palatable oral liquid antibiotics, and (iii) suggestions to overcome these challenges.

### 3.3 Theme 1: Challenges associated with prescribing and dispensing oral liquid antibiotic for children

The theme of challenges of prescribing and dispensing oral liquid antibiotic for paediatric patients included the factors affecting prescribing and dispensing antibiotics, reasons to prescribe or dispense poorly palatable antibiotics, palatability discussions with parents, and factors affecting oral liquid antibiotic administration.

#### 3.3.1 Subtheme: Factors affecting prescribing and dispensing antibiotics

Identified factors included clinical guidelines, availability of antibiotics, and age, weight and allergy status of the child. Clinical guidelines were considered mainly by GPs as they make the initial decision to select an antibiotic for a paediatric patient. Availability of antibiotics was highlighted by both professions as a recent challenge that they had to consider. Pharmacists reported that shortage of antibiotics caused them to manipulate solid dosage forms into liquid. Age and weight were factors that both professions considered to calculate the antibiotic dose. Both professions considered allergy status of the child before prescribing or dispensing an oral liquid antibiotic.

*“First of all, when I prescribe for children, the first questions I should ask the parents about is allergy. If the child is allergic to penicillin, so I have to prescribe something of [a] different family such as clarithromycin and my first choice in this case is usually Klacid*® [clarithromycin].” (GP5).

“*Because of the shortage with antibiotics at the moment, there is a leaflet going around with how to make the solid dosage forms into liquid format, but It's just that's effort in trying to make sure that you're getting the right dose [and] you're open to error then.”* (Ph1).

#### 3.3.2 Subtheme: Reasons for prescribing and dispensing poorly palatable antibiotics

All participants reported that in some circumstances they had to select an unpalatable antibiotic even though they know that it has poor palatability. The main reasons reported were the type of infection, lack of alternatives, and allergy.

“*If I had a swab or culture back with a specific sensitivity, then I really would try to encourage them to find some way that they can get the child to take it.*” (GP3).

“*I'm more confined in terms of the lack of alternatives, so I would just have to go with what we have in stock.*” (Ph10).

“*If there is a penicillin allergy, I have to prescribe macrolide, so I prescribe Klacid*® *[clarithromycin] though I know it is unpalatable but unfortunately you are limited.*” (GP5).

#### 3.3.3 Subtheme: Discussion of palatability with the parents

This subtheme encompasses the extent of antibiotic palatability is discussed with parents, and reasons for not initiating a discussion about palatability with parents.

##### 3.3.3.1 Extent of palatability discussion with parents

All participants reported that they only consider discussing oral liquid antibiotic palatability if a parent raises the issue. They all stated that they never initiate the palatability conversation with parents. However, participants reported that parents often negotiated about antibiotic palatability with both professions such as asking for alternative if they struggled to administer a previous antibiotic to the child. The parent might also ask for an alternative antibiotic even before completing the original antibiotic course.

“*I wouldn't discuss palatability unless the parents specifically asked, I would just say the label directions.”* (Ph2).

*“I suppose I changed when the parent, a day or two later, were saying, look, we've tried and get however many doses in, and it hasn't happened. So, could we try [an] alternative?” (GP1).*

##### 3.3.3.2 Reasons for not initiating the discussion about palatability with parents

Participants reported different reasons for not starting the palatability discussion with parents. The main reasons were to avoid negotiation because they do not have alternatives; they avoid raising the potential issue until the child tries to take the antibiotic. They also avoid raising palatability issues with parents in case parents pass this fact to the child who will then be primed to refuse the antibiotic.

*“I think if you warn the parent that it's not nice, they're going to probably pass that on to the child before they've even started. Just try and give it [antibiotic] to them.”* (Ph9).

#### 3.3.4 Subtheme: Formulation factors affecting oral liquid antibiotic acceptability

Participants reported that the factors affecting oral liquid antibiotic administration by parents were frequency, volume, and palatability. Palatability factors included taste, smell, and texture. Texture involved grittiness and viscosity. Participants stated that it is a challenge for the parents to give antibiotics to children, in particular if it required a high number of doses per day, a large volume, or poor palatability. Participants reported the poor acceptability of flucloxacillin formulations due to their poor taste, unpleasant smell and four times a day dosing schedule. The clarithromycin product Klacid was also reported as poorly palatable due to grainy texture. A list of formulation acceptability issues associated with the poorly palatable oral liquid antibiotics reported by the participants are outlined in Table 1.

*“It depends on how many times per day, some antibiotics has the advantage that it can be taken twice per day, parents forget about the doses if it is more than that.” (GP5).*

**Table 1:** Reported antibiotic acceptability issues detailing antibiotics and brands where mentioned.

Antibiotic	Brands mentioned	Acceptability issues
Flucloxacillin	–	Taste, smell, frequency
Clarithromycin	Klacid	Texture
Phenoxymethylpenicillin	Calvapen	Flavour^a^, texture
Trimethoprim	–	Taste
Co-amoxiclav	Augmentin	Flavour
Azithromycin	Zithromax	Flavour, texture
Cephalexin	Keflex	Taste
Amoxicillin	Pinamox	Flavour
Benzyl penicillin (Penicillin G)	–	Taste

a Flavour is the combined perception of mouthfeel, texture, taste and smell.

### 3.4 Theme 2: Impact of prescribing or dispensing poorly palatable oral liquid antibiotics

The second main theme was the impact of prescribing or dispensing poorly palatable oral liquid antibiotics. This encompassed two sub-themes: patient impact, and health system impact.

#### 3.4.1 Subtheme: Patient impact

The patient impact subtheme explored the impact on children and their parents of prescribing and dispensing practices relating to unpalatable oral liquid antibiotics. This included parents' attempt to overcome palatability issues, administration of inaccurate doses, and stress at home.

##### 3.4.1.1 Parents' attempt to overcome palatability issues

Lack of parent counselling about how to administer a poorly palatable antibiotic to their child in a safe manner can cause the parents to use unlicensed dose manipulation practices. Participants reported that poor palatability can lead to unlicensed manipulation of the antibiotic by parents by mixing the dose with food or drink. Examples were given by participants of particularly challenging situations that they encountered. Examples include a participant who reported that a grandmother intended to use nebulizers to give an oral antibiotic formulation to a child because of its poor palatability. Another example was participant who stated that a parent misled her by saying that their child had a penicillin allergy in order to avoid taking an unpalatable antibiotic they have been dispensed before. Participants believed that it is important to discuss palatability with parents to build a trust relationship with the parents as these examples show.

*“I had a scary thing recently actually, that there was a grandmother of the child. She was looking at nebulizers and it turned out when I was talking to her then that she was going to try and put the liquid antibiotic into the nebulizer and see if she could give the antibiotic to the child that way, because the child just wouldn't take the antibiotic..” (Ph10).*

*“The mom also told me she had a penicillin allergy. So, the choice is then very limited, and then the doctor said oh no, they [the child] actually don't have penicillin allergy, they just don't like the taste of all the penicillin ones. …. then I told the mom that there's almost nothing left on the shelf besides penicillin antibiotics. Then the mom was like, well, she's not really allergic. She's …. got the pink one last time and the pink one was just horrible. She doesn't want the pink one again, do you have another colour? So, I just went to the shelf and looked at the colours of them and I was like, yeah, I have a yellow and a white one and the doctor on the phone said, yeah, perfect one of them. This is just such a bad way of deciding what antibiotic to give someone, but they were happy.” (Ph12).*

##### 3.4.1.2 Inaccurate dose

Participants reported that parents manipulating antibiotics because of poor palatability could lead to administration of an underdose. Parents try to disguise either the consistency or the flavour of the original product. They thought it is better to try and give the child part of the dose rather than taking nothing at all.

*“I suppose if the parent is mixing it with a bit of whatever it might be, you kind of feel they would take little. They might not get the full dose.”* (Ph3).

##### 3.4.1.3 Stress at home

Participants reported that parents sometimes want to give their child the antibiotic by forcing it into the child. Forcing a child to take a poorly palatable oral liquid antibiotic can cause stress at home. Participants described that a parent's job is so much more difficult when the antibiotic is unpalatable. A pharmacist reported that her own children have to be held down by her husband in order to give them some of the antibiotic dose.

*“I've just finished a course of Pinamox [amoxicillin] with them, where they roared and screamed no for the yellow one and for the course of the antibiotic. I've had to get my husband to catch and hold of one of them and it's like trying to dose a calf. So yeah, it's not easy. I think from a psychological point of view for kids, it's not great that they have to be held down nearly to have an antibiotic.”* (Ph1).

#### 3.4.2 Subtheme: Health system impact

This subtheme explored the influence of prescribing and dispensing unpalatable oral liquid antibiotics on the health system. This comprised antimicrobial resistance and increased wastage.

##### 3.4.2.1 Antimicrobial resistance

Participants reported that they are trying to follow the clinical guidelines to avoid contributing to antimicrobial resistance. However, poor palatability can cause them to select a non-first line antibiotic to make sure that the child will take it.

*“Kids especially kids who'd have special needs, their parent would want daily dosing antibiotic. I do have a patient where the doctor will give Klacid [clarithromycin] because it will be a smaller quantity and you'll be able to get it into them easier which isn't great for antibiotic resistance, but it just comes down to palatability. You just have to stand your ground when it comes to that situation with the way that everything is coming now with antibiotic resistance and that it's going to come to the stage where nothing is going to work if we give in to my patients. It's not a sweet chocolate. You go with the guidelines for a reason.” (Ph1).*

##### 3.4.2.2 Increased wastage

Participants informed that poor palatability of oral liquid antibiotics can increase wastage when the parents asked for a different antibiotic. Parents would recognize the antibiotic once reconstituted based on their previous experience. However, sometimes they ask for alternative after trying it and before completing the antibiotic course. In all cases, the unused antibiotic has to be disposed of in the pharmacy.

*“You'd have something to dispense, and then you bring it out to the parents, they would then tell you we don't want this one because the child doesn't like the taste. That is something that happened. Maybe it's something we should consider earlier on in the process of dispensing rather than later, so that it doesn't come to the point where the antibiotic is made-up ready to go on the fridge. It might need to be a little bit earlier rather than one of the last things I would consider.” (Ph4).*

### 3.5 Theme 3: Suggestions to overcome challenges

The third theme was suggestions to overcome challenges. This included two sub-themes: counselling parents while prescribing and before dispensing the antibiotic and increasing awareness of antibiotic palatability among health care professionals.

##### 3.5.1.1 Subtheme: Discussion with parents while prescribing and before dispensing

Participants highlighted the importance of increasing parental awareness about the reasons for selecting the first line antibiotic and encourage the parent to attempt to administer it to the child. They emphasised that it is crucial to discuss palatability issues with parents during prescribing and before dispensing to avoid non-disclosure and unlicensed manipulation practices by parents. This could be accomplished by counselling parents and supporting them through the provision of advice about safe and effective methods of administration. Pharmacists reported that they provide syringes to place the dose into the side of the child's mouth to get rid of the dose very quickly instead of a spoon that washes the dose around the mouth. Some participants reported that they advise parents to bribe the children and give them a reward after taking a dose. However, participants preferred provision of approved information on manipulation techniques by including such information in the patient information leaflet. Participants would also welcome guidelines on counselling parents on administering unpalatable oral liquid antibiotics. Furthermore, many participants reported that parents referred to an antibiotic by its colour, rather than by the drug or product name, a practice that can create a barrier to effective communication about the antibiotic.

*“Parents associate antibiotics with the colour to find out if what they're getting this time is the same as what they got last time.” (Ph7).*

*“At least knowing what the flavour is of an oral liquid antibiotic for a child and knowing the colour and knowing the consistency of it and perhaps before reconstituting the antibiotic to ask the parents or the person collecting the medicine, you know, this is the one we have in stock, it's a suspension, it's yellow in colour, it is lemon flavoured. Is that OK to dispense for the child. So, they perhaps will be additional questions to be added to the checklist before dispensing.” (Ph11).*

##### 3.5.1.2 Subtheme: Increasing awareness of palatability among healthcare professionals

Many participants reported that they have never tasted an oral liquid antibiotic themselves. There is a lack of knowledge about antibiotic palatability issues in both professions, in particular a lack of flavour awareness among GPs. Therefore, it important to increase their awareness about this issue to be able to support parents.

*“I have no idea what it tastes like” (Ph12).*

*“I don't know what is the pink one. What is the yellow one? What is the white one? I'm not really familiar with them” (GP6).*

## 4 Discussion

Through qualitative interviews with pharmacists and GPs, the key findings of this study identify specific challenges related to prescribing and dispensing poorly palatable oral liquid antibiotics, and the impact of these challenges on both patients and the healthcare system. The study findings also provide healthcare professionals suggestions for overcoming these challenges. These findings build upon earlier studies focused on physicians' perceptions of prescribing inappropriate antibiotics based on parents' expectations^21^, ^22^, 23. by focusing on the impact of antibiotic poor palatability. The findings also expand on prior studies which identified antibiotics with poor palatability^10^^,^^24^^,^^25^ exploring the impact of poor palatability on prescribing or dispensing practices, and related patient and health system impacts.

Clinical guidelines, availability of antibiotics, and age, weight, and allergy status of the child were the main factors affecting the choice of antibiotics for children by both professions. However, the type of infection, lack of alternatives, and allergy influence healthcare professionals to select an oral liquid antibiotic with poor palatability. In the present study penicillin allergy was highlighted as a factor that could limit choice of antibiotics. It was reported in previous studies that patients, both adults and children, with a medical history of penicillin allergy had increased risk of receiving broad-spectrum antibiotics compared to non-penicillin allergic patients.^21^, ^22^, 23. In the current study, clarithromycin was reported as an appropriate alternative antibiotic in the case of penicillin allergy. However, the study also revealed that some oral liquid formulations of clarithromycin are unpalatable for children because of a grainy texture. This issue was reported in previous studies.^10^^,^^24^

Formulation factors that affect paediatric acceptability of oral liquid antibiotics identified in this study were reported to be frequency of administration, dose volume, and palatability. Likewise, Liu et al. reported that the acceptability of oral liquid formulations was directly influenced by the same factors.^25^ Flucloxacillin was highlighted as an unacceptable oral liquid antibiotic in the present study because of its poor taste, unpleasant smell, and high dose frequency. Likewise, Venables et al. and Elgammal et al. reported the unacceptable taste and smell of flucloxacillin.^1^^,^^10^ Lack of palatable oral liquid antibiotic alternatives confined healthcare professionals' selection of antibiotics. Although oral liquid dosage form is the preferred formulation among young children, there is a lack of availability of palatable options.^26^ Klingmann et al. reported the lack of appropriate antibiotic formulations for use in hospitalised children^27^ and Ventola has highlighted that the lack new antibiotics developed in recent years has limited the antibiotic options available to treat bacterial infection.^28^

However, the current study highlights how the shortage of paediatric antibiotic formulations leads to manipulating solid dosage form into liquid dosage form for children. This manipulation can affect dose accuracy.^29^ The European Medicines Agency highlighted the need for age-appropriate medicines for children.^30^ Formulation of antibiotic minitablets that are suitable for children would overcome this problem. Recent studies have demonstrated the acceptability of minitablets among young children aged 6 months and above.^31^^,^^32^ Münch et al. showed high acceptability of 2.0 mm and 2.5 mm diameter film-coated placebo mini-tablets in young children aged between 6 months and 6 years.^31^ Acceptability in the study was assessed based on swallowability. The overall palatability of minitablets was observed pleasant or neutral by over 90 % of the children based on observing the child's immediate physical reactions after formulation administration. A potential advantage of a minitablet dosage form for children is guaranteed administration of the accurate medication doses.

This study highlights the differences between GPs, pharmacists, and parents' experiences regarding oral liquid antibiotics knowledge. Parents often associate antibiotics by the colour of the formulation. However, GPs reported that they are unfamiliar with antibiotic product colour or are only aware through parent comments. Although pharmacists know the colour of different antibiotics, both professions reported that they have never tasted the antibiotic formulations. Bradshaw et al. reported that a tasting test of liquid formulations changed physicians' prescribing preferences for some infectious conditions.^33^ The physicians in that study switched from prescribing amoxicillin to cefdinir for otitis media and from azithromycin to amoxicillin for pneumonia. Raising awareness of formulation characteristics such as taste, smell, texture, and colour of different antibiotic formulations is needed among healthcare professionals to be able to select the most appropriate formulation for each child. The current study emphasizes the need of updates in prescribing and dispensing guidelines as well as including training programmes for both GPs and pharmacists with a particular focus on understanding the challenges around oral liquid antibiotic palatability for paediatric patients. Training provision can highlight how poor palatability can influence treatment adherence and the potential clinical repercussions of non-completion of treatments, including antimicrobial resistance.

Non-adherence to antibiotic treatment because of poor palatability can lead to switching to a more palatable antibiotic which is typically a more broad-spectrum antibiotic.^9^^,^^10^The findings of this study highlight that the poor palatability of oral liquid antibiotics can lead to prescribing a non-first line antibiotic which could contribute to antimicrobial resistance. Additionally, non-adherence to antibiotic treatment due palatability could also lead to an increase in the development of antimicrobial-resistance.^34^, ^35^, ^36^ Antimicrobial resistance is a global problem that threatens global public population.^11^^,^^37^^,^^38^ Therefore, there is a need to improve adherence to regimes of oral first-line antibiotic formulations by improving their palatability.

The study findings revealed that oral liquid antibiotics poor palatability can lead to unlicensed manipulation by parents and the administration of inaccurate dose. Participants in this study reported that parents manipulate poorly palatable antibiotics through mixing the required dose with food or drink. However, unlicensed manipulation can affect stability, efficacy and bioavailability of medicines and should be avoided.^16^ The current study identified the need for pharmacists to discuss and give suitable administration advice to parents based on the specific characteristics of each dispensed antibiotic formulation, its palatability and its associated challenges prior to dispensing. This may help to avoid manipulation of doses and non-disclosure of unlicenced dosing practices by parents. The current study asserted the need for the development of manipulation guidelines for paediatric medicines by authorities such as medicines and healthcare regulators. This finding aligns with earlier research.^29^

The current study highlighted that GPs and pharmacists avoid discussion with parents about oral liquid antibiotic palatability. Such lack of discussion with parents was similarly highlighted in previous studies.^39^^,^^40^ Barden et al. conducted a focus group study with parents and physicians to study their perceptions about antibiotic use.^40^ The study reported that lack of discussion with parents was a reason for antibiotic overuse. The physicians in the study reported that prescribing antibiotics was influenced by their perception that parents expected to receive an antibiotic prescription during the medical visit. However, parents revealed that they would be satisfied if the physician explained the reasons for not prescribing an antibiotic. Similarly, Mangione et al.'s cross-sectional study to examine the relationships between physician and parents revealed that a communication gap between physicians and parents increases physicians' perceptions that antibiotics are expected by parents and leads to inappropriate antibiotic prescribing.^41^ Counselling guidelines are needed to improve communication between healthcare professionals and parents about antibiotic usage and in particular about include information regarding oral liquid antibiotic palatability.

Conversation with parents about palatability, as well as the importance of receiving the first-choice antibiotic during prescribing and before dispensing, were identified as approaches that may improve adherence to the antibiotic treatment. Participants reported parents negotiate regarding palatability with both professions by asking for an alternative antibiotic if they struggled to administer a previous antibiotic to the child. They might also ask for an alternative even before completing the antibiotic course. Previous studies report that deviation from clinical guidelines by GPs or switching between antibiotic brands by pharmacists because of poor palatability was often based on parents' requests.^10^^,^^17^ Incomplete antibiotic treatment because of poor palatability increases medicines wastage. The current study revealed that providing parent with information regarding the colour and flavour of the selected antibiotic before reconstitution could decrease wastage by avoiding switching antibiotic brands after the initial formulation being reconstituted. Bergene and colleagues have previously recommended that discussion with parents about availability of different formulations would lead to dispensing the most suitable antibiotic product for every child.^17^ Proactive dialogue discussing antibiotic palatability with parents and carers during the prescribing process and prior to dispensing can reinforce parents and carers' understanding of the importance of following the prescribed treatment, despite potential palatability issues, and minimise switching from first choice antibiotics treatments.

In summary study findings highlight a need to develop more palatable oral liquid formulations of the first-line antibiotics. It is envisaged that developing more palatable oral liquid antibiotics, either through flavouring, texture enhancement or innovative formulation approaches such as minitablets, will contribute significantly to improving paediatric treatment adherence. Future studies are suggested to investigate the acceptability of different formulation approaches, as well as the evaluation of education and training strategies to overcome barriers related to palatability in different social and cultural contexts.

## 5 Strengths and limitations

This qualitative study included interviews with both GPs and pharmacists. The research team included members of both professions. Six participants were parents themselves who provided their experience as healthcare professionals as well as parents. A strength of the study was the broad range of years of experience of participants, ranging from 2 to 27 years with an average experience of 11 years, which provided the views and experiences of a diverse representation of practitioners. While the participants with more years of experience have more experience of this topic, those with less experience brought a fresh perspective to the problem. The study was limited to exploring palatability issues from the experience of GPs and pharmacists. Based on the findings of this study, a follow-on study with other primary and secondary care health professionals, parents, and children would be worthwhile to determine how the themes identified relate to their experiences.

A limitation of the study was the lower number of GP participants, almost half the number of pharmacists. The reason for the lower GP number can be related to the overall difficulty in engaging GPs in research internationally despite their critical role in extending knowledge and translating new information into practice.^42^, ^43^, ^44^ Despite the relatively small GP sample size, a range of clear views were obtained from which it was possible to identify valuable themes.

There may a perceived element of potential bias among volunteers recruited by research team's professional networks. However, as healthcare professionals describing experiences and challenges faced during routine practice this was not considered a significant limitation. Conducting the interviews via Microsoft Teams may have limited potential participants from participating in the research. Limitations such as technical issues, planning, privacy and rapport related to the use of online video conferencing for conducting qualitative studies has been recently reported.^45^ However, for this study interviews were conducted via videoconferencing due to the advantages it offers in terms of convenience and time flexibility for the interviewees, with the ability to conduct interviews outside normal business hours. In general, interview studies may be subject to interviewer or social desirability biases. The study comprised semi-structured interviews to ensure adequate predictive validity. In addition, participants' willingness to share their experiences or perspectives can be impacted by the interviewer's communication style. To minimise these biases, the interviews were conducted by two members of the research team and samples of transcripts were independently reviewed and coded by three members of the research team.

Most of the participants were recruited by research team's professional networks in the Cork region which may limit the generalisability of the study, however many findings are supported by the broader literature.

## 6 Conclusion

There is a need to increase palatability awareness among healthcare professionals and parents of children prescribed oral liquid antibiotics. Raising palatability awareness among healthcare professionals will enable them to deliver appropriate counselling to parents. Discussion with parents about palatability and the importance of receiving the first-choice antibiotic should occur during prescribing and before dispensing. This may decrease the wastage through avoiding switching antibiotic brands after reconstitution. The development of more palatable antibiotic formulations, either through flavour or texture enhancement or innovative formulations such as minitablets, may play a role in improving medicine taking practices in children.

## Funding

This publication has emanated from research supported in part by a research grant from 10.13039/501100001602Science Foundation Ireland (SFI) and is co-funded under the 10.13039/501100008530European Regional Development Fund [grant number 12/RC/2275(P2)].

## CRediT authorship contribution statement

**Ayat Elgammal:** Writing – original draft, Visualization, Methodology, Investigation, Formal analysis, Data curation, Conceptualization. **Joseph Ryan:** Writing – review & editing, Methodology, Investigation. **Colin Bradley:** Writing – review & editing, Methodology, Conceptualization. **Abina Crean:** Writing – review & editing, Validation, Supervision, Project administration, Methodology, Funding acquisition, Conceptualization. **Margaret Bermingham:** Writing – review & editing, Validation, Supervision, Project administration, Methodology, Data curation, Conceptualization.

## Declaration of competing interest

The authors declare that they have no conflict of interest.

## Data Availability

Data are available and can be furnished upon reasonable request to the corresponding author.
