# Supplementary material for: Challenges prescribing and dispensing oral antibiotics with poor palatability for paediatric patients: A qualitative interview study with GPs and pharmacists
Source: Explor Res Clin Soc Pharm. 2024 Nov 23;17:100546. doi: 10.1016/j.rcsop.2024.100546 (PMC11647503; doi:10.1016/j.rcsop.2024.100546)
Supplement: Supplementary material [file mmc1.docx]

**Supplementary File 1:** Interview Topic Guides

**GPs and trainee GPs**

1. Which factors do you consider when prescribing oral liquid antibiotics for children?

2. How do you decide which drug / brand to prescribe?

3. To what extent could poor palatability affect your prescription?

4. How are you made aware that an oral liquid antibiotic is poorly palatable for a child?

5. In what manner could discussions with parents/ caregivers about antibiotic palatability and their willingness to give it to their children change your antibiotic prescribing?

6. What advice would you give to a parent/ caregiver who is unable to administer an unpalatable oral liquid antibiotic to children?

7. Which oral liquid antibiotics do you think are more palatable/ unpalatable to children? Why do you think they are palatable/unpalatable?

8. Under what circumstances would you prescribe oral liquid antibiotic with poor palatability?

9. Having completed this interview, to what extent has it raised your awareness of palatability issues when prescribing?

10. Having completed this interview, to what extent will change your practice with regards to prescribing antibiotics for children?

11. Is there anything else you would like to add?

**Pharmacists**

1. Which factors do you consider when selecting a prescribed oral liquid antibiotic product for children?

2. How do you decide which drug formulation/ brand to dispense?

3. To what extent could poor palatability affect your choice of product to dispense?

4. How are you made aware that an oral liquid antibiotic is poorly palatable for a child?

5. In what manner could discussions with parents/ caregivers about antibiotic palatability and their willingness to give it to their children change your antibiotic dispensing practice?

6. What advice would you give to a parent/ caregiver who is unable to administer an unpalatable oral liquid antibiotic to children?

7. Which oral liquid antibiotics do you think are more palatable/ unpalatable to children? Why do you think they are palatable/ unpalatable?

8. Under what circumstances would you dispense an oral liquid antibiotic with poor palatability?

9. Having completed this interview, to what extent has it raised your awareness of palatability issues when dispensing?

10. Having completed this interview, to what extent will change your practice with regards to dispensing antibiotics for children?

11. Is there anything else you would like to add?
